# Bacterial Hyaluronidase Promotes Ascending GBS Infection and Preterm Birth

**DOI:** 10.1128/mBio.00781-16

**Published:** 2016-06-28

**Authors:** Jay Vornhagen, Phoenicia Quach, Erica Boldenow, Sean Merillat, Christopher Whidbey, Lisa Y. Ngo, K. M. Adams Waldorf, Lakshmi Rajagopal

**Affiliations:** a Department of Global Health, University of Washington, Seattle, Washington, USA; b Seattle Children’s Research Institute, Seattle, Washington, USA; c Department of Pediatric Infectious Diseases, University of Washington, Seattle, Washington, USA; d Department of Obstetrics and Gynecology, University of Washington, Seattle, Washington, USA

## Abstract

Preterm birth increases the risk of adverse birth outcomes and is the leading cause of neonatal mortality. A significant cause of preterm birth is *in utero* infection with vaginal microorganisms. These vaginal microorganisms are often recovered from the amniotic fluid of preterm birth cases. A vaginal microorganism frequently associated with preterm birth is group B streptococcus (GBS), or *Streptococcus agalactiae*. However, the molecular mechanisms underlying GBS ascension are poorly understood. Here, we describe the role of the GBS hyaluronidase in ascending infection and preterm birth. We show that clinical GBS strains associated with preterm labor or neonatal infections have increased hyaluronidase activity compared to commensal strains obtained from rectovaginal swabs of healthy women. Using a murine model of ascending infection, we show that hyaluronidase activity was associated with increased ascending GBS infection, preterm birth, and fetal demise. Interestingly, hyaluronidase activity reduced uterine inflammation but did not impact placental or fetal inflammation. Our study shows that hyaluronidase activity enables GBS to subvert uterine immune responses, leading to increased rates of ascending infection and preterm birth. These findings have important implications for the development of therapies to prevent *in utero* infection and preterm birth.

## IMPORTANCE

GBS are a family of bacteria that frequently colonize the vagina of pregnant women. In some cases, GBS ascend from the vagina into the uterine space, leading to fetal injury and preterm birth. Unfortunately, little is known about the mechanisms underlying ascending GBS infection. In this study, we show that a GBS virulence factor, HylB, shows higher activity in strains isolated from cases of preterm birth than those isolates from rectovaginal swabs of healthy women. We discovered that GBS rely on HylB to avoid immune detection in uterine tissue, but not placental tissue, which leads to increased rates of fetal injury and preterm birth. These studies provide novel insight into the underlying mechanisms of ascending infection.

## INTRODUCTION

Preterm birth is a major indicator for neonatal morbidity and mortality (1, 2). Approximately 6 to 15% of all deliveries are preterm, resulting in an estimated 4 million neonatal deaths per year, making preterm birth the leading cause of mortality in neonates and in children under 5 years of age (3–7). The largest burden of neonatal and under-5 mortality due to preterm birth is concentrated in sub-Saharan Africa and southern Asia, where health care systems are often too weak to effectively manage high preterm birth rates (8). Preterm birth rates are also alarmingly high in the developed world, including North America, where preterm birth has an annual health care cost in the tens of billions of dollars (2, 9). In order to reduce the burden and subsequent cost of preterm birth, we need a better understanding of the causes and physiology of its biological processes.

Although the clinical events associated with preterm birth have been well studied, its underlying causes remain ill defined. An estimated 25 to 40% of preterm births are a result of *in utero* bacterial infection (10). Bacteria can be recovered from the amniotic fluid of preterm birth cases (11–13). Bacteria reach the amniotic fluid by means of ascending infection, which occurs when bacteria penetrate the cervical barrier and enter the uterus (2). Once in the uterine space, bacteria cause multiple physiological events associated with preterm birth, including increased levels of proinflammatory cytokines, chorioamniotic membrane rupture, cervical ripening, and uterine contraction (2, 11, 14–16). One group of bacteria associated with these physiological events that has been recovered from amniotic fluid is group B streptococcus (GBS), or *Streptococcus agalactiae* (12, 14, 15, 17, 18). GBS are a leading cause of neonatal morbidity and mortality, and approximately 30% of healthy women are rectovaginally colonized with GBS (3–5, 7). Heavy vaginal GBS colonization is the primary risk factor for GBS-associated preterm birth (19, 20). Despite the large number of women at risk for GBS-associated preterm birth, little is known about the bacterial and host factors involved in GBS colonization and ascending infection.

Multiple host and bacterial factors play a role in ascending infection and preterm birth. One such factor is the high-molecular-weight polymer hyaluronic acid, which is cleaved by hyaluronidases. Hyaluronic acid polymers have multiple roles, including a structural role in epithelial cell extracellular matrix (ECM) formation, aiding in cell migration, cell-cell signaling, and induction of ECM remodeling enzymes and inflammation (21). Recently, it has been shown that cervical hyaluronic acid protects against ascending infection and preterm birth due to its role in epithelial barrier function and that lipopolysaccharide-induced murine cervical hyaluronidase expression increases preterm birth rates (22–24). These studies highlight the importance of hyaluronic acid and hyaluronidases during pathogen colonization and preterm birth, but a mechanistic connection between pathogen hyaluronidase activity during vaginal colonization and preterm birth is not known.

Interestingly, GBS produces a hyaluronidase (here referred to as “HylB”), encoded by the *hylB* gene. HylB was first identified in the 1950s and is well characterized as a specific exolytic enzyme (25–27). It was recently determined that HylB plays an important role in GBS evasion of the host immune system (28). These studies show that GBS degrades hyaluronic acid into disaccharide fragments, which blocks Toll-like receptors (TLRs) 2 and 4, preventing GBS ligands from activating proinflammatory signaling cascades (28). Despite these exciting advances, it is unknown if HylB is important for ascending GBS infection and/or preterm birth.

Here, we show that clinical GBS strains isolated from women in preterm labor had increased levels of HylB activity compared to commensal strains isolated from rectogvaginal swabs of pregnant women not in labor. Using a mouse model of ascending GBS infection, we observed that genetic ablation of *hylB* in GBS leads to decreased rates of bacterial ascension and fetal demise. Finally, we show that maternal uterine cells infected with wild-type (WT) GBS exhibit a lower level of inflammation compared to cells infected with GBS lacking HylB. These data are the first to describe a role for the GBS hyaluronidase in ascending infection and preterm birth.

## RESULTS

### Clinical GBS isolates associated with invasive disease exhibit increased hyaluronidase activity.

**FIGURE 1:**
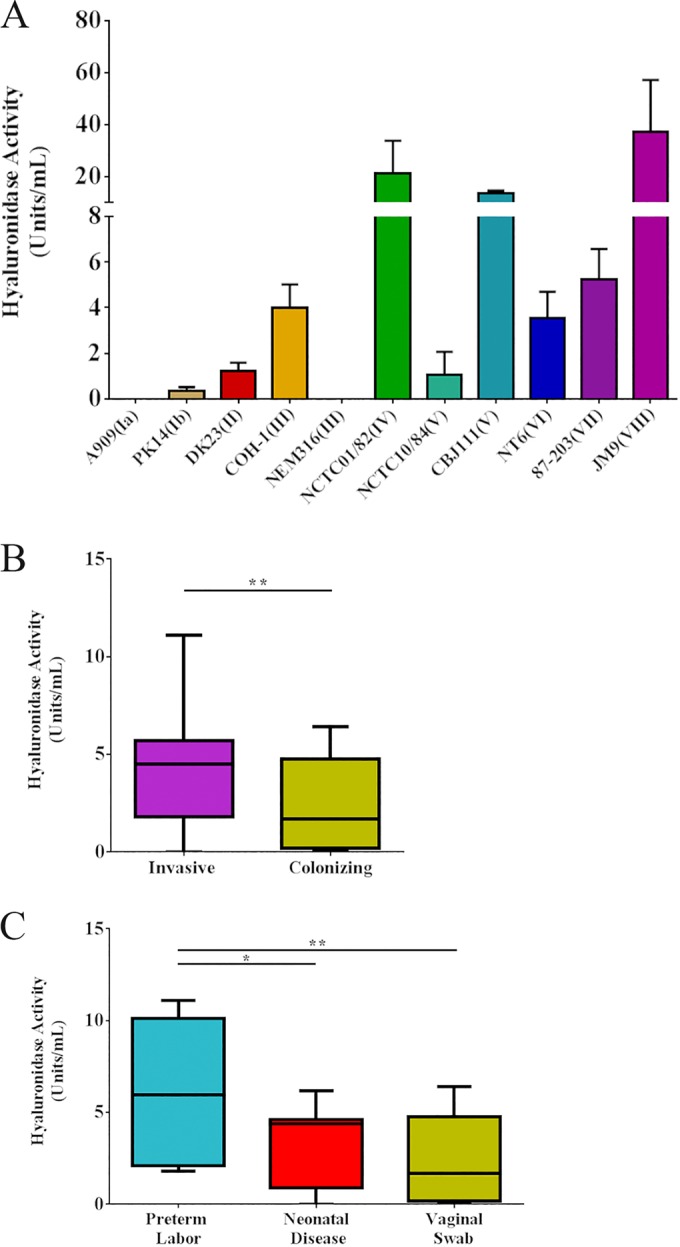
Disease-associated clinical GBS isolates display increased hyaluronidase activity. GBS strains representative of each capsular serotype (A) and clinical GBS strains from invasive disease (*n* = 23) or from rectovaginal swabs (*n* = 48) (B) were grown overnight and assessed for their hyaluronidase activity. Invasive clinical GBS strains were also stratified into those isolated from women undergoing preterm labor (*n* = 8) or those isolates from neonatal disease (*n* = 15) (C). All experiments were performed three independent times in triplicate. Unpaired Student’s *t* test was used to assess statistical significance between single groups (B [**, *P* > 0.01]). In panel A, data bars display the mean ± standard error of the mean SEM, while panels B and C show box plots with the mean, 25% percentile, 75% percentile, and error bars indicating minimum and maximum values. Tukey’s multiple comparison test following one-way ANOVA was used to assess statistical significance between multiple groups (C [*, *P* > 0.05; **, *P* > 0.01]).

We hypothesized that GBS-encoded hyaluronidase activity may be critical for ascending infection and preterm birth. To measure HylB activity in various GBS isolates, we adapted a hyaluronidase activity assay that was previously described (29). Among our laboratory collection of GBS strains representing each capsular serotype, we observed that strains showed various levels of hyaluronidase activity. GBS strains A909 (serotype Ia) and NEM316 (serotype III) showed little to no activity, whereas strains NCTC 01/82 (serotype IV), CBJ111 (serotype V), and JM9 (serotype XIII) showed the highest levels of activity (Fig. 1A). Interestingly, strains belonging to the same capsular serotype exhibited various levels of hyaluronidase activity (compare COH1 to NEM316 and NCTC 10/84 to CBJ111). These data suggest that while hyaluronidase activity varied among GBS strains, it is not correlated to capsular serotype.

We next sought to compare hyaluronidase activities in clinical GBS strains isolated from amniotic fluid or neonatal blood (invasive infection, *n* = 23) to those obtained from women who were rectovaginally colonized with GBS without symptoms of invasive infection (*n* = 48). Overall hyaluronidase activity was higher in GBS strains obtained from invasive settings compared to that of commensal settings (Fig. 1B). Finally, we stratified invasive GBS strains into those isolated from neonatal disease without preterm birth (*n* = 15) and GBS-associated preterm birth (*n* = 8). Interestingly, isolates from cases of GBS-associated preterm birth displayed modestly higher levels of hyaluronidase activity compared to isolates from neonatal disease and much higher levels of activity compared to isolates from vaginal swabs (Fig. 1C). Taken together, these data suggest a role for HylB in invasive GBS disease and preterm birth.

### HylB promotes ascending GBS infection and adverse birth outcomes.

**FIGURE 2:**
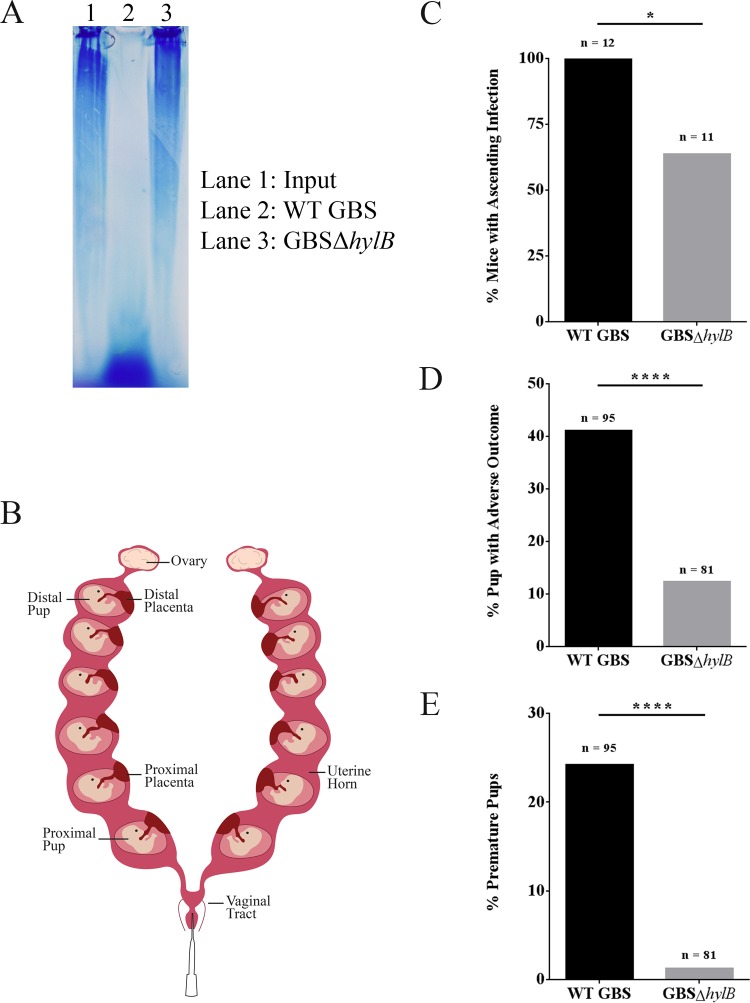
HylB activity leads to increased rates of ascending infection and adverse pregnancy outcomes, including preterm birth. Hyaluronic acid incubated with or without GBS was analyzed by agarose gel electrophoresis. The WT GBS strain COH1 shows increased hyaluronidase activity (lane 2, WT GBS) compared to the isogenic *hylB* mutant (lane 3, GBSΔ*hylB*), which shows no hyaluronidase activity (compare to lane 1, input [A]). Pregnant female C57BL/6J mice (a schematic can be found in panel B) were inoculated with approximately 10^8^ CFU of WT GBS (*n* = 12) or GBSΔ*hylB* (*n* = 11). Contingency analyses were performed on observed outcomes of these mice. Observed outcomes were ascending bacterial infection (C), pups with adverse outcome (either born prematurely or with *in utero* fetal death [D]), and pups born prematurely (E). Fisher’s exact test was used to assess statistical significance between groups (A to C [*, *P* > 0.05; ****, *P* > 0.00005]).

Given the higher levels of hyaluronidase activity in GBS strains isolated from women in preterm labor, we examined if HylB activity is important for ascending infection and preterm birth. We adapted a pregnant murine model of ascending GBS infection, recently developed by Randis and colleagues (30), with the modification that we omitted the use of gelatin during vaginal inoculation of pregnant mice. We constructed a *hylB* mutant (here referred to as GBSΔ*hylB*) in WT GBS COH1 (serotype III, ST17 clone associated with increased virulence [31]) and confirmed the loss of hyaluronidase activity in the GBSΔ*hylB* mutant by gel electrophoresis. The results shown in Fig. 2 indicate that WT GBS COH1 displays high hyaluronidase activity (indicated by the lower hyaluronic acid band in Fig. 2A, lane 2), whereas the GBSΔ*hylB* strain displays no hyaluronidase activity (Fig. 2A, lane 3).

**FIGURE 3:**
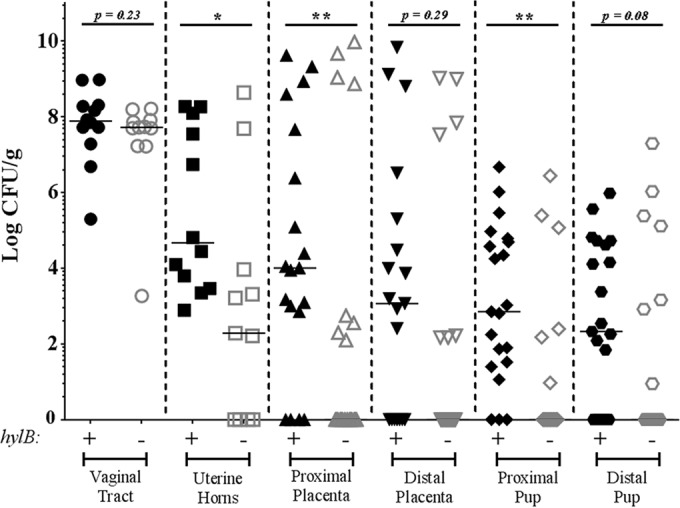
HylB activity leads to increased bacterial ascension. Pregnant female C57BL/6J mice were inoculated with approximately 10^8^ CFU of WT GBS (*n* = 12) or GBSΔ*hylB* (*n* = 11). The vaginal tract, uterine horns, distal and proximal placentas, and distal and proximal pups of these mice were removed and assessed for bacterial load by serial dilution plating. Data are displayed as individual data points with median bars. Mann-Whitney *U* test was used to assess statistical significance between groups (*, *P* > 0.05; **, *P* > 0.01).

Next, we vaginally inoculated pregnant C57BL/6J mice on day 15 of pregnancy (E15) with approximately 10^8^ CFU of WT GBS or isogenic GBSΔ*hylB* (Fig. 2B). The mice were then monitored for signs of preterm birth (defined as vaginal bleeding or pups in cage) for up to 72 h postinoculation. Upon signs of preterm birth or at 72 h postinoculation (whichever occurred first), the mice were euthanized, a midline laparotomy was performed, and *in utero* fetal death (IUFD) was recorded prior to subsequent analysis. Intriguingly, mice inoculated with WT GBS showed significantly higher rates of ascending infection and adverse pregnancy outcomes (preterm birth and/or IUFD) compared to mice inoculated with GBSΔ*hylB* (Fig. 2C to E). Consistent with these findings, pregnant mice inoculated with WT GBS also exhibited significantly higher bacterial load in the uterine horns compared to mice inoculated with GBSΔ*hylB* (Fig. 3). Placentas and pups most proximal to the vaginal tract showed significantly more bacterial CFU in mice inoculated with WT GBS than in mice inoculated with GBSΔ*hylB*. Similar trends were also seen in the distal pups and placenta, but these data did not reach statistical significance. Notably, vaginal colonization did not appear to be significantly different between mice inoculated with WT GBS and those inoculated with GBSΔ*hylB*, suggesting that the differences in infection outcomes cannot be attributed to differences in vaginal colonization. Additionally, there is a weak, but statistically significant, correlation between the amount of ascended GBS load (average of uterine horn, pup, and placental CFU) and the percentage of pups with an adverse birth outcome (see Fig. S1 in the supplemental material). Together, these data show that HylB plays an important role in ascending GBS infection leading to adverse birth outcomes.

### Hyaluronidase (HylB) activity dampens uterine immune responses during ascending GBS infection.

**FIGURE 4:**
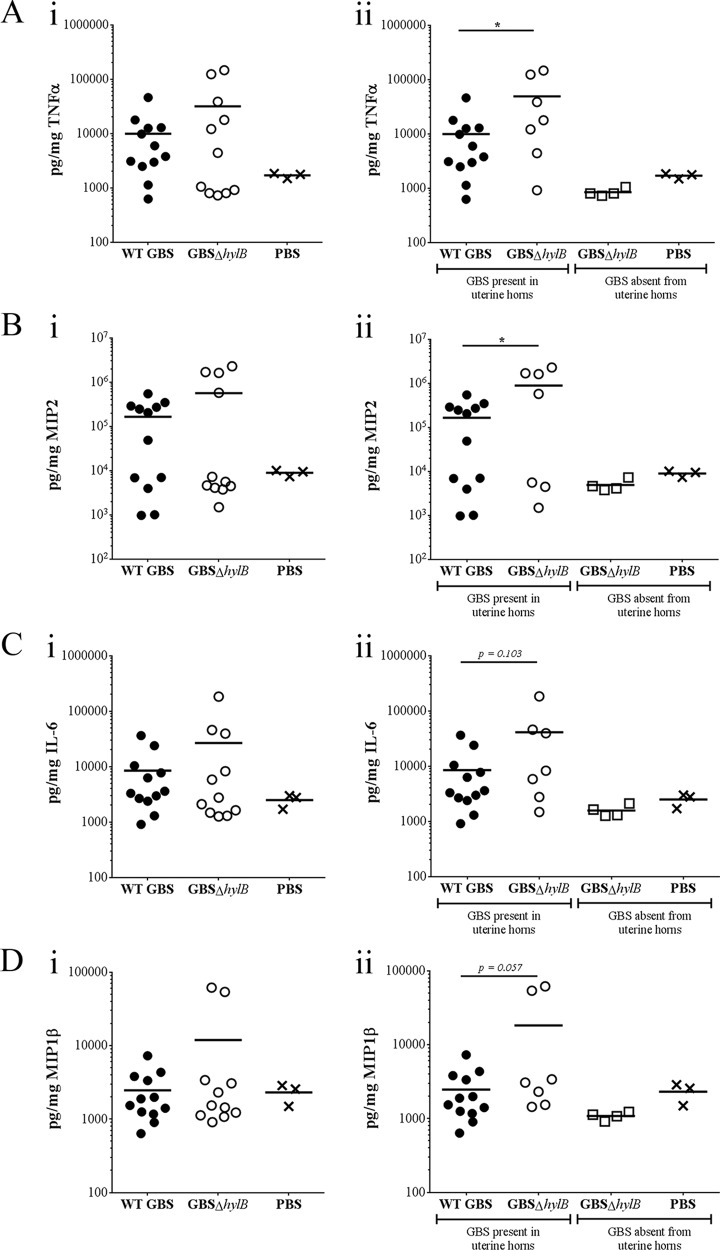
GBS HylB activity blocks uterine inflammation *in vivo*. Luminex assays were used to assess the levels of the inflammatory markers TNF-α (A), MIP2 (B), IL-6 (C), and MIP1β (D) in the uterine tissues of pregnant female C57BL/6J mice inoculated with approximately 10^8^ CFU of WT GBS strain COH1 (*n* = 12), GBSΔ*hylB* (*n* = 11), or PBS (*n* = 3). Data are represented as immune responses in uterine samples irrespective of bacteria (Ai to Di) or as uterine samples with GBS to those without GBS (either inoculated with GBS or PBS [Aii to Dii]). Unpaired Student’s *t* test was used to assess statistical significance between groups (A to D [*, *P* > 0.05]). Data bars display the mean.

Previous studies by Kolar et al. have shown that GBS utilizes the *hylB*-encoded hyaluronidase to degrade hyaluronic acid into disaccharide fragments, which blocks TLR2/4 signaling to prevent inflammation (28). Uterine epithelial cells express high levels of TLRs 1, 2, and 6, which are more responsive to Gram-positive bacteria than to other types of pathogens (32). Additionally, uterine TLR2 expression in mice increases as gestation progresses, whereas placental and fetal TLR2 expression decreases during gestational progression (33). To determine if the GBS hyaluronidase mediates uterine immune responses during ascending infection, we performed Luminex assays to measure levels of inflammatory cytokines in uterine tissues from pregnant mice vaginally inoculated with WT GBS or GBSΔ*hylB* (Fig. 4Ai to Di). We found that inflammatory cytokines from mice inoculated with GBSΔ*hylB* clustered into discrete low and high groups that correlated with the presence or absence of bacteria. Therefore, we stratified the uterine samples to those with bacteria and noted significantly higher levels of tumor necrosis factor alpha (TNF-α) and macrophage inflammatory protein 2 (MIP2) (Fig. 4Aii and Bii) and modestly higher levels of interleukin-6 (IL-6) and MIP1β (Fig. 4Cii and Dii) in the uterine tissues of mice inoculated with GBSΔ*hylB* compared to that of mice inoculated with WT GBS. Levels of inflammatory markers in the bacterium-free uterine tissues (all from mice inoculated with GBSΔ*hylB*) were similar to that of mice inoculated with phosphate-buffered saline (PBS) (Fig. 4Aii to Dii). No differences were observed in levels of IL-10, IL-1β, or GROα in uterine tissues (see Fig. S2 in the supplemental material), nor were there differences in the levels of TNF-α, MIP2, IL-6, MIP1β, IL-10, IL-1β, or GROα in placental or fetal tissues (see Fig. S3 in the supplemental material). These data suggest that immune responses in uterine tissues, rather than placental or fetal tissues, are responsible for decreasing the incidence of ascending GBS infection and that GBS circumvents this response by blocking TLRs through HylB activity.

### Hyaluronidase activity dampens uterine immune responses, but not placental immune responses, in human tissues.

Given our *in vivo* findings, we set out to determine if HylB modulates inflammatory responses in human tissues. We first used an *ex vivo* model of GBS infection of chorioamniotic membranes. Intact chorioamniotic membranes were collected from healthy women not in labor undergoing caesarean sections at term and were mounted on Transwells as previously described (15). Membranes were then inoculated with approximately 10^7^ CFU on the choriodecidual/maternal side, which was supplemented with 1.25 mg/ml hyaluronic acid. At 4 and 24 h postinfection, medium was collected from both the chorionic and amniotic sides of the membranes. TNF-α, IL-6, and IL-8 concentrations were measured by enzyme-linked immunosorbent assay (ELISA). As was observed *in vivo*, GBS induced significant cytokine expression from the gestational membranes, but this inflammation was not decreased by HylB (see Fig. S4 in the supplemental material). These data indicate that HylB does not play a role in dampening the inflammatory response from the placenta and suggest that another tissue is responsible for preventing ascending infection.

**FIGURE 5:**
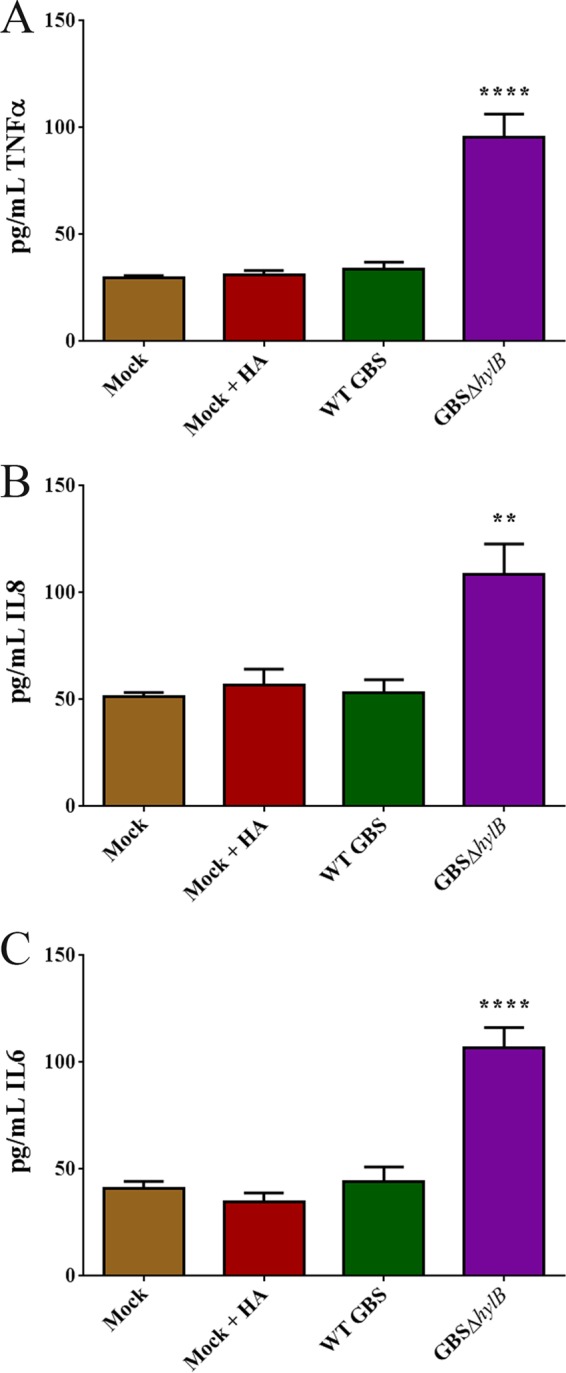
HylB activity dampens immune responses in immortalized endometrial cells. ELISAs were used to assess the levels of TNF-α (A), IL-8 (B), and IL-6 (C) in immortalized endometrial HEC-1-B cells that were infected for 48 h with WT GBS or GBSΔ*hylB* at an MOI of 0.01 in media supplemented with 1.25 mg/ml hyaluronic acid (HA). Experiments were performed in triplicate and repeated six times. One-way ANOVA with Tukey’s multiple correction test was used to assess statistical significance between groups (A to C). Asterisks over single bars indicate significant compared to all other groups (**, *P* > 0.005; ****, *P* > 0.00005).

We then tested the impact of HylB in uterine tissue using an immortalized human endometrial cell (HEC-1-B) model of infection. HEC-1-B cells were infected with GBS at a multiplicity of infection (MOI) of 0.01 in media supplemented with 1.25 mg/ml hyaluronic acid. Cell culture supernatant was collected at 48 h postinfection, and levels of TNF-α, IL-6, and IL-8 were measured by ELISA. Interestingly, HEC-1-B cells infected with GBS lacking *hylB* display significantly higher levels of inflammatory cytokines TNF-α, IL-6, and IL-8 than WT GBS, which corroborates our *in vivo* findings (Fig. 5). These data suggest a role for uterine immune responses in preventing ascending infection by clearing GBS that have entered the uterine space. GBS that are able to block uterine immune responses through HylB appear to be able to disseminate into deeper tissues, such as placental and fetal tissues, leading to adverse pregnancy outcomes such as *in utero* fetal demise and/or preterm birth. Collectively, our studies indicate the importance of GBS-encoded hyaluronidase in suppression of uterine immune responses during ascending infection and preterm birth.

## DISCUSSION

The work presented in this article establishes a novel role for the GBS hyaluronidase, HylB, as a critical virulence factor in ascending GBS infection and resulting preterm birth. We have shown that GBS strains isolated from women undergoing preterm labor show higher levels of hyaluronidase activity and that this activity permits ascending infection by reducing antibacterial inflammation in uterine tissue. Recent work has described the role of HylB in tissue dissemination and immune evasion during septic GBS infection, but the role of HylB in ascending infection has heretofore been unstudied (28, 34). Our data indicate that HylB is important for pathogenesis during ascending infection. GBSΔ*hylB* showed decreased bacterial ascension and fetal demise and induced more inflammation in both human and murine uterine tissues. Moreover, as hyaluronidases encoded by other bacterial pathogens, such as *Streptococcus pyogenes* and *Staphylococcus aureus*, are critical for virulence (35, 36), our work will be relevant to understanding the role of microbial hyaluronidases during disease pathogenesis.

Hyaluronic acid plays a pivotal role in the progression of pregnancy and labor (22–24, 37, 38). During parturition, hyaluronic acid production drastically increases in cervical tissue until it comprises approximately 1.0% of the dry weight of the cervix (23). For a successful vaginal birth, the cervix must undergo a softening process (referred to as cervical ripening) in order to allow cervical distention (39). Cleavage of hyaluronic acid is vital for proper cervical ripening. Humans and mice produce multiple hyaluronidases to cleave the abundance of hyaluronic acid present in the cervix, and application of exogenous hyaluronidase is one method used to induce cervical ripening and labor (40). Interestingly, vaginal inoculation with hyaluronidase-encoding *Escherichia coli* leads to increased preterm birth rates in mice (22). These data suggest that microbial hyaluronidases can induce cervical ripening and labor, and our data support this hypothesis. While we do not know that the GBS hyaluronidase specifically induces cervical ripening, we show that it is crucial in increasing GBS infection-associated preterm birth (Fig. 2D and E). The ability of the GBS hyaluronidase, and other microbial hyaluronidases, to induce cervical ripening needs to be explored further. Inhibition of microbial hyaluronidases may be a possible avenue for developing therapeutics to reduce preterm birthrates.

**FIGURE 6:**
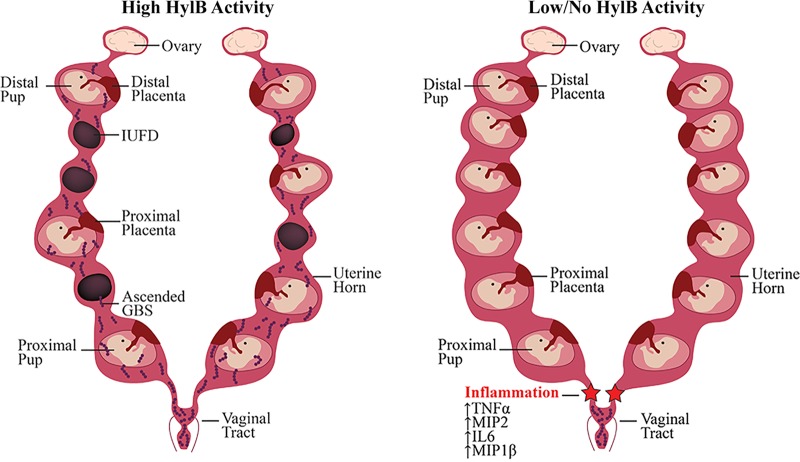
Proposed model of HylB-mediated preterm birth. GBS colonizes the rectovaginal tract of 20 to 40% of women, where it behaves as a commensal organism. GBS strains with high hyaluronidase (HylB) activity are able to ascend into the uterine space by blocking uterine TLR signaling and avoiding immune detection. Once in the uterine space, ascended GBS are able to invade placental and fetal tissues, leading to *in utero* fetal demise and/or preterm birth. GBS strains with low HylB activity levels are not able to subvert immune detection and are either unable to ascend into the uterine space or conversely are cleared by immune cells soon after ascension. These strains are relegated to the vaginal tract and thus are not able to infect fetal and placental tissues or induce preterm birth.

Traditionally, inflammation is thought to be a driver of preterm labor, so it is counterintuitive that genetic ablation of *hylB* leads to more uterine inflammation but less preterm birth. We speculate that this is due to the temporal nature of uterine inflammation. The uterine epithelium expresses high levels of the bacteria-responsive TLRs 1, 2, and 6 (32). Moreover, TLR2 expression increases in the uterine tissue during gestational progression, while TLR2 expression declines in other gestational tissues, such as placental tissue and chorionic membranes (33). We reason that the uterus is the primary immunological barrier that prevents Gram-positive bacterial invasion of gestational tissues. If this immunological barrier fails due to inhibited TLR signaling, ascended bacteria may avoid detection by the host. This may allow dissemination into placental and fetal tissues and preterm birth, as was observed in pregnant mice inoculated with WT GBS (Fig. 2C and 3). Conversely, if TLR signaling is not inhibited, an increase in uterine inflammation may lead to bacterial clearance, such as what we observed in pregnant mice inoculated with GBSΔ*hylB* (Fig. 2C, 3A, and 4A to D). The timing and magnitude of inflammation may also play important roles in preterm labor as an outcome of ascending bacterial infection. If the increase in uterine inflammation is a transient event, rather than a sustained event, the host may be able to clear the infection without the induction of labor. It is interesting that mice inoculated with GBSΔ*hylB* that did not show ascending infection also had lower levels of inflammation than those that did show ascending infection (Fig. 4). We hypothesize that ascended bacteria caused a large spike in inflammation early in infection, leading to their clearance from the uterine space. Once the bacteria are cleared, the inflammation may rapidly dissipate, promoting healthy pregnancy (Fig. 6). These results imply a need for earlier GBS screening during pregnancy, but additional research is needed to fully address this hypothesis.

Preterm birth is the leading cause of adverse pregnancy outcomes for both the mother and fetus, and a significant number of preterm births are due to *in utero* infection with vaginal flora, such as GBS (1, 2, 4, 6–8, 14). Understanding the virulence factors that allow vaginal flora to switch from commensal colonizers to ascending pathogens is crucial for developing successful interventions to prevent *in utero* infection and resulting preterm birth. While studies have characterized the role of the GBS hemolytic pigment in vaginal colonization, ascending infection, induction of inflammation, and fetal damage (30, 41, 42), we know little about the role of other GBS virulence factors involved in these processes. By defining the mechanism underlying these different virulence factors, it may be possible to develop strategies for intervention. Interestingly, multiple compounds are able to inhibit microbial hyaluronidase activity (36, 43). To our knowledge, little attention has been given to finding a hyaluronidase inhibitor specific to GBS, but such an inhibitor may be an effective means of combating multiple types of GBS infections.

We have described a novel role for the GBS hyaluronidase in ascending infection and preterm birth. First, we draw a strong association between GBS hyaluronidase activity and serious disease outcomes in clinical isolates derived from human samples. Next, we show that the presence of the GBS hyaluronidase permits increased bacterial ascension from the vaginal tract to the uterine space, which is associated with a reduction in uterine inflammation and leukocyte invasion compared to GBS lacking the hyaluronidase. Finally, we are suggesting a more refined role for uterine inflammation during pregnancy, wherein inflammation can potentially lead to the elimination of ascended bacteria without inducing preterm labor. Future research should focus on further defining the role of the GBS hyaluronidase in these processes and developing a hyaluronidase inhibitor to prevent ascending GBS infections.

## MATERIALS AND METHODS

### Ethics statement.

All animal experiments were approved by the Seattle Children’s Research Institutional Animal Care and Use Committee (protocol 13907) and performed in strict accordance with the recommendations in the *Guide for the Care and Use of Laboratory Animals, 8th ed.* (44).

### Bacterial strains.

The chemicals in this study were purchased from Sigma Aldrich unless stated otherwise. The WT GBS strain COH1 used in these studies is a serotype III (sequence type 17 [ST17] clone) clinical isolate from an infected human newborn (45). GBS cultures were grown in tryptic soy broth (TSB) or on tryptic soy agar (TSA) (Difco Laboratories) at 30 or 37°C in 5% CO_2_ and monitored at 600 nm. The *hylB* allelic replacement mutant was generated using methods previously described (46). Briefly, 1.0-kb regions flanking either side of the *hylB* gene (*hylB* 3′ forward, CCA AGG AGC CTA AAG AGG CCT GAC CCA AGA GAT TAA C; *hylB* 3′ reverse, TTT AGC CAT TTT TAC TCC TTA GGT TTT AAA ATT GTA AAC; *hylB* 5′ forward, ATT GTT TTA GCT AAC CGT ACA TAA AAA ACC TAT C; *hylB* 5′ reverse, GTA GGC GCT AGG GAC AAC TGT CCT TGA TAA ATT GAC) and a kanamycin resistance cassette (Kan^r^ forward, GGA GTA AAA AAT GGC TAA AAT GAG AAT ATC AC; Kan^r^ reverse, ACG GTT AGC TAA AAC AAT TCA TCC AGT AAA ATA TAA TAT TTT ATT TTC) were PCR amplified from genomic DNA (for flanking regions) or from the plasmid pCIV2 (for the Kan^r^ cassette). Each primer contained a short (15- to 25-bp) region of homology that corresponded to either the temperature-sensitive cloning vector pHY304 or the next corresponding region. The pHY304 cloning vector was PCR linearized (pHY304 forward, GTC CCT AGC GCC TAC GGG; pHY304 reverse, CTC TTT AGC TCC TTG GAA GCT GTC), and all four fragments (2 flanking regions, Kan^r^ cassette, and linear pHY304) were ligated using the Gibson Assembly cloning kit (New England Biolabs). This plasmid was then electroporated into the WT GBS strain COH1, and selection for allelic replacement was performed as previously described (46).

### Clinical isolates.

GBS clinical isolates were collected as previously described (15, 41). Briefly, rectovaginal swabs were obtained from women in their third trimester of pregnancy at the University of Washington Medical Center and Harborview Medical Center in 2007 under University of Washington IRB 30308; samples were collected without any identifiers or clinical information, and a waiver for written informed consent was obtained for testing anonymous samples. GBS clinical isolates from amniotic fluid, chorioamnion, and/or cord blood were obtained from women enrolled with preterm labor and intact membranes at less than or equal to 34 weeks of gestation at the University of Washington Medical Center, Swedish Medical Center, and Virginia Mason Medical Center, Seattle, Washington between 25 June 1991 and 30 June 1997. This cohort was previously described, the University of Washington Institutional Review Board approved the study protocol, and all participants provided written informed consent (18). Fifteen GBS isolates from infected newborns were kindly provided by Sharon Hillier, University of Pittsburgh.

### Human cell culture.

Human HEC-1-B endometrial cells (ATCC strain HTB-113) were grown at 37°C in 5% CO_2_. Cells were maintained in Eagle’s minimal essential medium (MEM [Corning]), supplemented with 10% heat-inactivated fetal bovine serum (Corning) and 50 to 100 IU/ml penicillin and 50 to 100 µg/ml streptomycin (Corning). Cells were split every 3 to 4 days and passaged at a 1:4 dilution. Twenty-four hours prior to infection, antibiotic-containing medium was aspirated, cells were washed with sterile PBS, and medium was replaced with antibiotic-free, serum-free MEM. At the time of infection the medium was aspirated and replaced with fresh antibiotic-free, serum-free medium supplemented with or without 1.25 mg/ml hyaluronic acid. All GBS infections were performed at an approximate MOI of 0.01 for 48 h between passages 7 and 14.

### Hyaluronidase activity assay.

GBS strains were grown overnight in TSB at 30°C with 5.0% CO_2_. Overnight cultures were pelleted at 4,000 rpm for 8 min, and the resulting supernatants were collected for analysis. Fifty microliters of spent supernatants was prewarmed for 5 to 10 min at 37°C with 5.0% CO_2_ and then was added to 200 µl hyaluronic acid solution (1.25 mg hyaluronic acid sodium salt from rooster comb [Sigma] dissolved in 36.0 mg/ml monobasic sodium phosphate [pH 5.35] at 37°C) and incubated at 37°C with 5.0% CO_2_ for 45 min. After incubation, 50 µl of 0.8 M sodium tetraborate (pH 9.1) preheated to 95°C was added, and the sample was boiled at 95°C for 3 min. Following boiling, 1.5 ml of 1.0% (wt/vol) 4-methylaminobenzaldehyde dissolved in 15.3 M acetic acid and 1.25 M HCl was added to the sample, and 200 µl was removed and read on a plate reader at 585 nm. To calculate the amount of hyaluronidase activity, standard curves of commercial hyaluronidase (hyaluronidase from bovine testes; Sigma) dissolved in TSB were created and included in conjunction with every assay. Hyaluronidase activity values from unknown samples were interpolated from the standard curve, which was fit to a 4-parameter logistic curve using GraphPad Prism 6 (La Jolla, CA). If sample reads exceeded the standard curve, the initial spent supernatant was diluted in TSB as necessary to fit within the standard curve range.

### Hyaluronic acid gel electrophoresis.

For hyaluronic gel electrophoresis, overnight cultures of GBS wild-type and GBSΔ*hylB* cells were pelleted at 4,000 rpm for 8 min, and then 50 µl of supernatant was incubated with 200 µl of hyaluronic acid solution (see above) for 45 min at 37°C with 5.0% CO_2_. After incubation, samples were boiled at 95°C for 3 min, frozen, and lyophilized until completely dry. Samples were then mixed with 17 µl of loading buffer (0.02% [wt/vol] bromophenol blue, 2 M sucrose in 1× Tris-borate-EDTA [TBE]) and resolved on a 0.8% agarose gel. Electrophoresis was carried out at a voltage of 100 V for approximately 2.5 h. Once the run was completed, the gel was placed in 500 ml of 0.005% Stains-All (Sigma) solution dissolved in 50% ethanol overnight under light-protected cover at room temperature and destained in water for ~1 h until the background was photobleached.

### Murine model of ascending GBS infection.

Six- to-8-week-old female C57BL/6J mice were obtained from The Jackson Laboratory and used for ascending infection studies. Female mice were individually paired with male C57BL/6J mice for 2 days and then separated and monitored for 14 days postseparation. Pregnancy was confirmed by observable weight gain and palpation for the presence of pups. Mice were inoculated on day 15 of pregnancy (E15). Overnight GBS cultures were subcultured 1:20, grown to optical density at 600 nm (OD_600_) of 0.3, pelleted at 4,000 rpm for 8 min, washed once with sterile PBS, and resuspended in sterile PBS to a final concentration of 10^10^ CFU/ml. Mice were anesthetized using 4% isoflurane, and 10 µl (~10^8^ CFU) of inoculum was administered into the vaginal tract using a micropipette. Mice were left inverted for 5 additional minutes under anesthesia and then returned to their cages and monitored until ambulatory. Mice were monitored twice daily up to 72 h postinoculation for signs of preterm birth (vaginal bleeding and/or pups in the cage). At 72 h postinfection, or earlier if preterm birth was observed, mothers were euthanized and a midline laparotomy was performed to identify fetal injury and loss of pregnancy and to collect maternal and fetal tissues. Tissues were excised, homogenized, and serially plated on TSB to determine the number of CFU associated with maternal and fetal tissues. All data were normalized to total tissue weight in grams. Homogenized tissue was then incubated overnight at 4°C in lysis buffer (0.15 M NH_4_Cl, 1 mM NaHCO_3_ [pH 7.2]) and pelleted, and supernatants were collected for further analysis as described below.

### Luminex assays of murine tissues.

Tissue lysates from the mouse ascending infection model (see “Murine model of ascending GBS infection” above) were thawed and centrifuged at 10,000 × *g* for 5 min at 4°C to remove residual cell debris. Fifty microliters of the supernatants was then used for cytokine analysis (IL-10, IL-1β, IL-6, GROα, TNF-α, MIP2, and MIP1β) by Luminex assay (Procartaplex Multiplex immunoassay; eBioscience) as per the manufacturer’s instructions. Expected cytokine concentrations for the standard curve were translated into logarithmic scale and fit to a 5-parameter logistic curve, and median fluorescence intensity readings from unknown samples were then interpolated from this standard curve using GraphPad Prism 6 (La Jolla, CA). These data were translated out of logarithmic scale back into concentration values (picograms per milliliter) and were then normalized to total tissue weight in milligrams.

### Cytokine analysis in GBS-infected human chorioamnion.

Human chorioamniotic/placental membranes were collected from deidentified healthy term pregnancies undergoing scheduled caesarean sections at the University of Washington Hospital. Since identifiable information was not collected, this research is exempt from IRB review (see letter from University of Washington IRB, determination form 44282, “Use of discarded placentas”). Chorioamniotic membranes were cultured on Transwells as previously described (15). Briefly, membranes were dissected from placenta immediately following delivery and transported on ice. Membranes were rinsed in PBS and mounted on Transwell inserts (12 mm) lacking the synthetic filter membrane. Gestational membranes were held in place with sterile latex bands. Transwells were placed in 12-well plates with 0.5 ml medium (Dulbecco’s minimal essential medium [DMEM] with l-glutamine supplemented with 1% fetal bovine serum [FBS] and penicillin/streptomycin) in the upper chamber and 1.5 ml medium in the lower chamber at 37 C. Following overnight acclimation, membranes on Transwell inserts were washed and inoculated with approximately 10^7^ CFU in the upper (choriodecidual/maternal) chamber in DMEM supplemented with or without 1.25 mg/ml hyaluronic acid. Medium was sampled at 4 and 24 h from both the apical and basal compartments and stored at −20°C until analysis. ELISAs for cytokine levels were purchased from R and D Systems and performed as per the manufacturer’s instruction. Medium from infected tissue was diluted 1:50 (TNF-α) or 1:100 (IL-6, IL-8, and CCL4), and medium from control tissue was diluted 1:4.

### Statistical analysis.

The Mann-Whitney test, Fisher’s exact test, or Tukey’s multiple comparison test following analysis of variance (ANOVA) was used to estimate differences as appropriate, and a *P* value of <0.05 was considered significant. Statistics were performed using GraphPad Prism version 5.0 for Windows (GraphPad Software, La Jolla, CA).

## SUPPLEMENTAL MATERIAL

Figure S1  Correlation between ascended GBS and preterm birth. Shown are Spearman’s correlation between the number of ascended GBS (average CFU in uterine space, placentas, and pups) and the percentage of preterm pups (either in the cage or IUFD). Download Figure S1, PDF file, 0.1 MB

Figure S2  Inflammatory markers not affected by HylB in the uterine space. Luminex assays were used to assess the levels of the inflammatory markers IL-10 (A), IL-1β (B), and GROα (C) in the uterine tissues of pregnant female C57BL/6J mice inoculated with approximately 10^8^ CFU of COH1 (*n* = 12), COH1Δ*hylB* (*n* = 11), or PBS (*n* = 3). Data are shown from all 3 groups of uterine samples (Ai to Di), uterine samples with GBS present, or uterine samples without GBS (either inoculated with GBS or PBS [Aii to Dii]). Unpaired Student’s *t* test was used to assess statistical significance between groups (A to D [*, *P* > 0.05]). Download Figure S2, PDF file, 0.2 MB

Figure S3  Inflammation not affected by HylB in distal placental or distal pup tissues. Luminex assays were used to assess the levels of the inflammatory markers TNF-α (A), MIP2 (B), IL-6 (C), MIP1β (D), IL-10 (E), IL-1β (F), and GROα (G) in the distal placental tissues (see Fig. 2B for schematic) of pregnant female C57BL/6J mice inoculated with approximately 10^8^ CFU of COH1 (*n* = 5), COH1Δ*hylB* (*n* = 5), or PBS (*n* = 3). Download Figure S3, PDF file, 0.2 MB

Figure S4  Inflammation not affected by HylB in *ex vivo* gestational membranes. ELISAs were used to assess the levels of the inflammatory markers TNF-α (A), IL-6 (B), IL-8 (C), CCL4 (D) in the gestational tissues inoculated with approximately 10^7^ CFU of COH1 or COH1Δ*hylB*. Culture medium was supplemented with 1.25 mg/ml hyaluronic acid (HA). Experiments were performed in duplicate on at least 3 gestational tissues. One-way ANOVA with Tukey’s multiple correction test was used to assess statistical significance between groups (A to D [*, *P* > 0.05; **, *P* > 0.005; ***, *P* > 0.0005; ****, *P* > 0.00005]). LOD, limit of detection. Download Figure S4, PDF file, 0.3 MB

## References

[1] Liu L, Johnson HL, Cousens S, Perin J, Scott S, Lawn JE, Rudan I, Campbell H, Cibulskis R, Li M, Mathers C, Black RE, Child Health Epidemiology Reference Group of WHO, UNICEF 2012 Global, regional, and national causes of child mortality: an updated systematic analysis for 2010 with time trends since 2000. Lancet 379:2151–2161. doi:10.1016/S0140-6736(12)60560-1. 22579125

[2] Romero R, Dey SK, Fisher SJ 2014 Preterm labor: one syndrome, many causes. Science 345:760–765. doi:10.1126/science.1251816. 25124429 PMC4191866

[3] Katz J, Lee AC, Kozuki N, Lawn JE, Cousens S, Blencowe H, Ezzati M, Bhutta ZA, Marchant T, Willey BA, Adair L, Barros F, Baqui AH, Christian P, Fawzi W, Gonzalez R, Humphrey J, Huybregts L, Kolsteren P, Mongkolchati A, Mullany LC, Ndyomugyenyi R, Nien JK, Osrin D, Roberfroid D, Sania A, Schmiegelow C, Silveira MF, Tielsch J, Vaidya A, Velaphi SC, Victora CG, Watson-Jones D, Black RE 2013 Mortality risk in preterm and small-for-gestational-age infants in low-income and middle-income countries: a pooled country analysis. Lancet 382:417–425. doi:10.1016/S0140-6736(13)60993-9. 23746775 PMC3796350

[4] Lawn JE, Gravett MG, Nunes TM, Rubens CE, Stanton C, GAPPS Review Group 2010 Global report on preterm birth and stillbirth (1 of 7): definitions, description of the burden and opportunities to improve data. BMC Pregnancy Childbirth 10(Suppl 1):S1. doi:10.1186/1471-2393-10-S1-S1. 20233382 PMC2841772

[5] Blencowe H, Cousens S, Oestergaard MZ, Chou D, Moller AB, Narwal R, Adler A, Vera Garcia C, Rohde S, Say L, Lawn JE 2012 National, regional, and worldwide estimates of preterm birthrates in the year 2010 with time trends since 1990 for selected countries: a systematic analysis and implications. Lancet 379:2162–2172. doi:10.1016/S0140-6736(12)60820-4. 22682464

[6] Rubens CE, Gravett MG, Victora CG, Nunes TM, GAPPS Review Group 2010 Global report on preterm birth and stillbirth (7 of 7): mobilizing resources to accelerate innovative solutions (global action agenda). BMC Pregnancy Childbirth 10(Suppl 1):S7. doi:10.1186/1471-2393-10-S1-S7. 20233388 PMC2841775

[7] Slattery MM, Morrison JJ 2002 Preterm delivery. Lancet 360:1489–1497. doi:10.1016/S0140-6736(02)11476-0. 12433531

[8] Blencowe H, Cousens S, Chou D, Oestergaard M, Say L, Moller AB, Kinney M, Lawn J, Born Too Soon Preterm Birth Action Group 2013 Born too soon: the global epidemiology of 15 million preterm births. Reprod Health 10(Suppl 1):S2. doi:10.1186/1742-4755-10-S1-S2. 24625129 PMC3828585

[9] Patel RM, Kandefer S, Walsh MC, Bell EF, Carlo WA, Laptook AR, Sánchez PJ, Shankaran S, Van Meurs KP, Ball MB, Hale EC, Newman NS, Das A, Higgins RD, Stoll BJ, Eunice Kennedy Shriver National Institute of Child Health and Human Development Neonatal Research Network 2015 Causes and timing of death in extremely premature infants from 2000 through 2011. N Engl J Med 372:331–340. doi:10.1056/NEJMoa1403489. 25607427 PMC4349362

[10] Nold C, Anton L, Brown A, Elovitz M 2012 Inflammation promotes a cytokine response and disrupts the cervical epithelial barrier: a possible mechanism of premature cervical remodeling and preterm birth. Am J Obstet Gynecol 206:208.e1–208e 7. doi:10.1016/j.ajog.2011.12.036. 22285171

[11] Romero R, Gonzalez R, Sepulveda W, Brandt F, Ramirez M, Sorokin Y, Mazor M, Treadwell MC, Cotton DB 1992 Infection and labor. VIII. Microbial invasion of the amniotic cavity in patients with suspected cervical incompetence: prevalence and clinical significance. Am J Obstet Gynecol 167:1086–1091. doi:10.1016/S0002-9378(12)80043-3. 1415396

[12] Hillier SL, Krohn MA, Kiviat NB, Watts DH, Eschenbach DA 1991 Microbiologic causes and neonatal outcomes associated with chorioamnion infection. Am J Obstet Gynecol 165:955–961. doi:10.1016/0002-9378(91)90447-Y. 1951562

[13] Ueno T, Niimi H, Yoneda N, Yoneda S, Mori M, Tabata H, Minami H, Saito S, Kitajima I 2015 Eukaryote-made thermostable DNA polymerase enables rapid PCR-based detection of mycoplasma, ureaplasma and other bacteria in the amniotic fluid of preterm labor cases. PLoS One 10:e0129032. doi:10.1371/journal.pone.0129032. 26042418 PMC4456152

[14] Goldenberg RL, Hauth JC, Andrews WW 2000 Intrauterine infection and preterm delivery. N Engl J Med 342:1500–1507. doi:10.1056/NEJM200005183422007. 10816189

[15] Whidbey C, Harrell MI, Burnside K, Ngo L, Becraft AK, Iyer LM, Aravind L, Hitti J, Waldorf KM, Rajagopal L 2013 A hemolytic pigment of group B streptococcus allows bacterial penetration of human placenta. J Exp Med 210:1265–1281. doi:10.1084/jem.20122753. 23712433 PMC3674703

[16] Bastek JA, Gómez LM, Elovitz MA 2011 The role of inflammation and infection in preterm birth. Clin Perinatol 38:385–406. doi:10.1016/j.clp.2011.06.003. 21890015

[17] Hillier SL, Martius J, Krohn M, Kiviat N, Holmes KK, Eschenbach DA 1988 A case-control study of chorioamnionic infection and histologic chorioamnionitis in prematurity. N Engl J Med 319:972–978. doi:10.1056/NEJM198810133191503. 3262199

[18] Hitti J, Krohn MA, Patton DL, Tarczy-Hornoch P, Hillier SL, Cassen EM, Eschenbach DA 1997 Amniotic fluid tumor necrosis factor-alpha and the risk of respiratory distress syndrome among preterm infants. Am J Obstet Gynecol 177:50–56. doi:10.1016/S0002-9378(97)70437-X. 9240582

[19] Campbell JR, Hillier SL, Krohn MA, Ferrieri P, Zaleznik DF, Baker CJ 2000 Group B streptococcal colonization and serotype-specific immunity in pregnant women at delivery. Obstet Gynecol 96:498–503. 11004347 10.1016/s0029-7844(00)00977-7

[20] Allen U, Nimrod C, Macdonald N, Toye B, Stephens D, Marchessault V 1999 Relationship between antenatal group B streptococcal vaginal colonization and premature labour. Paediatr Child Health 4:465–469. 20212961 10.1093/pch/4.7.465 PMC2827758

[21] Stern R, Asari AA, Sugahara KN 2006 Hyaluronan fragments: an information-rich system. Eur J Cell Biol 85:699–715. doi:10.1016/j.ejcb.2006.05.009. 16822580

[22] Akgul Y, Word RA, Ensign LM, Yamaguchi Y, Lydon J, Hanes J, Mahendroo M 2014 Hyaluronan in cervical epithelia protects against infection-mediated preterm birth. J Clin Invest 124:5481–5489. doi:10.1172/JCI78765. 25384213 PMC4348952

[23] Akgul Y, Holt R, Mummert M, Word A, Mahendroo M 2012 Dynamic changes in cervical glycosaminoglycan composition during normal pregnancy and preterm birth. Endocrinology 153:3493–3503. doi:10.1210/en.2011-1950. 22529214 PMC3380303

[24] Mahendroo M 2012 Cervical remodeling in term and preterm birth: insights from an animal model. Reproduction 143:429–438. doi:10.1530/REP-11-0466. 22344465

[25] Baker JR, Pritchard DG 2000 Action pattern and substrate specificity of the hyaluronan lyase from group B streptococci. Biochem J 348:465–471. doi:10.1042/bj3480465. 10816443 PMC1221087

[26] Baker JR, Yu H, Morrison K, Averett WF, Pritchard DG 1997 Specificity of the hyaluronate lyase of group-B streptococcus toward unsulphated regions of chondroitin sulphate. Biochem J 327:65–71. doi:10.1042/bj3270065. 9355736 PMC1218764

[27] Gochnauer TA, Wilson JB 1951 Hyaluronidase production in vitro by streptococci isolated from bovine mastitis cases. Am J Vet Res 12:20–22. 14799725

[28] Kolar SL, Kyme P, Tseng CW, Soliman A, Kaplan A, Liang J, Nizet V, Jiang D, Murali R, Arditi M, Underhill DM, Liu GY 2015 Group B streptococcus evades host immunity by degrading hyaluronan. Cell Host Microbe 18:694–704. doi:10.1016/j.chom.2015.11.001. 26651945 PMC4683412

[29] Greif RL 1952 Colorimetric determination of hyaluronidase activity. J Biol Chem 194:619–625. 14927654

[30] Randis TM, Gelber SE, Hooven TA, Abellar RG, Akabas LH, Lewis EL, Walker LB, Byland LM, Nizet V, Ratner AJ 2014 Group B streptococcus beta-hemolysin/cytolysin breaches maternal-fetal barriers to cause preterm birth and intrauterine fetal demise in vivo. J Infect Dis 210:265–273. doi:10.1093/infdis/jiu067. 24474814 PMC4092248

[31] Musser JM, Mattingly SJ, Quentin R, Goudeau A, Selander RK 1989 Identification of a high-virulence clone of type III Streptococcus agalactiae (group B streptococcus) causing invasive neonatal disease. Proc Natl Acad Sci U S A 86:4731–4735. doi:10.1073/pnas.86.12.4731. 2660146 PMC287347

[32] Pioli PA, Amiel E, Schaefer TM, Connolly JE, Wira CR, Guyre PM 2004 Differential expression of Toll-like receptors 2 and 4 in tissues of the human female reproductive tract. Infect Immun 72:5799–5806. doi:10.1128/IAI.72.10.5799-5806.2004. 15385480 PMC517561

[33] Gonzalez JM, Xu H, Ofori E, Elovitz MA 2007 Toll-like receptors in the uterus, cervix, and placenta: is pregnancy an immunosuppressed state? Am J Obstet Gynecol 197:291.e1–296.e6. doi:10.1016/j.ajog.2007.06.021. 17826427

[34] Wang Z, Guo C, Xu Y, Liu G, Lu C, Liu Y 2014 Two novel functions of hyaluronidase from Streptococcus agalactiae are enhanced intracellular survival and inhibition of proinflammatory cytokine expression. Infect Immun 82:2615–2625. doi:10.1128/IAI.00022-14. 24711564 PMC4019169

[35] Hynes WL, Walton SL 2000 Hyaluronidases of Gram-positive bacteria. FEMS Microbiol Lett 183:201–207. doi:10.1111/j.1574-6968.2000.tb08958.x. 10675584

[36] Girish KS, Kemparaju K 2007 The magic glue hyaluronan and its eraser hyaluronidase: a biological overview. Life Sci 80:1921–1943. doi:10.1016/j.lfs.2007.02.037. 17408700

[37] Ruscheinsky M, De la Motte C, Mahendroo M 2008 Hyaluronan and its binding proteins during cervical ripening and parturition: dynamic changes in size, distribution and temporal sequence. Matrix Biol 27:487–497. doi:10.1016/j.matbio.2008.01.010. 18353623 PMC2492578

[38] Straach KJ, Shelton JM, Richardson JA, Hascall VC, Mahendroo MS 2005 Regulation of hyaluronan expression during cervical ripening. Glycobiology 15:55–65. doi:10.1093/glycob/cwh137. 15317739

[39] Maul H, Mackay L, Garfield RE 2006 Cervical ripening: biochemical, molecular, and clinical considerations. Clin Obstet Gynecol 49:551–563. doi:10.1097/00003081-200609000-00015. 16885662

[40] Kavanagh J, Kelly AJ, Thomas J 2006 Hyaluronidase for cervical ripening and induction of labour. Cochrane Database Syst Rev 2:CD003097. doi:10.1002/14651858.CD003097.pub2. PMC8691265 16625569

[41] Gendrin C, Vornhagen J, Ngo L, Whidbey C, Boldenow E, Santana-Ufret V, Clauson M, Burnside K, Galloway DP, Waldorf KA, Piliponsky AM, Rajagopal L 2015 Mast cell degranulation by a hemolytic lipid toxin decreases GBS colonization and infection. Sci Adv 1:e1400225. doi:10.1126/sciadv.1400225. 26425734 PMC4584422

[42] Whidbey C, Vornhagen J, Gendrin C, Boldenow E, Samson JM, Doering K, Ngo L, Ezekwe EA Jr, Gundlach JH, Elovitz MA, Liggitt D, Duncan JA, Adams Waldorf KM, Rajagopal L 2015 A streptococcal lipid toxin induces membrane permeabilization and pyroptosis leading to fetal injury. EMBO Mol Med 7:488–505. doi:10.15252/emmm.201404883. 25750210 PMC4403049

[43] Spickenreither M, Braun S, Bernhardt G, Dove S, Buschauer A 2006 Novel 6-O-acylated vitamin C derivatives as hyaluronidase inhibitors with selectivity for bacterial lyases. Bioorg Med Chem Lett 16:5313–5316. doi:10.1016/j.bmcl.2006.07.087. 16908142

[44] National Research Council 2011 Guide for the care and use of laboratory animals, 8th ed. National Academies Press, Washington, DC.

[45] Lancefield RC, McCarty M, Everly WN 1975 Multiple mouse-protective antibodies directed against group B streptococci. Special reference to antibodies effective against protein antigens. J Exp Med 142:165–179. doi:10.1084/jem.142.1.165. 1097573 PMC2189884

[46] Rajagopal L, Clancy A, Rubens CE 2003 A eukaryotic type serine/threonine kinase and phosphatase in Streptococcus agalactiae reversibly phosphorylate an inorganic pyrophosphatase and affect growth, cell segregation, and virulence. J Biol Chem 278:14429–14441. doi:10.1074/jbc.M212747200. 12562757

