# Supplementary figures and images for: Bacterial Hyaluronidase Promotes Ascending GBS Infection and Preterm Birth
Source: mBio. 2016 Jun 28;7(3):e00781-16. doi: 10.1128/mBio.00781-16 (PMC4937215; doi:10.1128/mBio.00781-16)

Figure S1

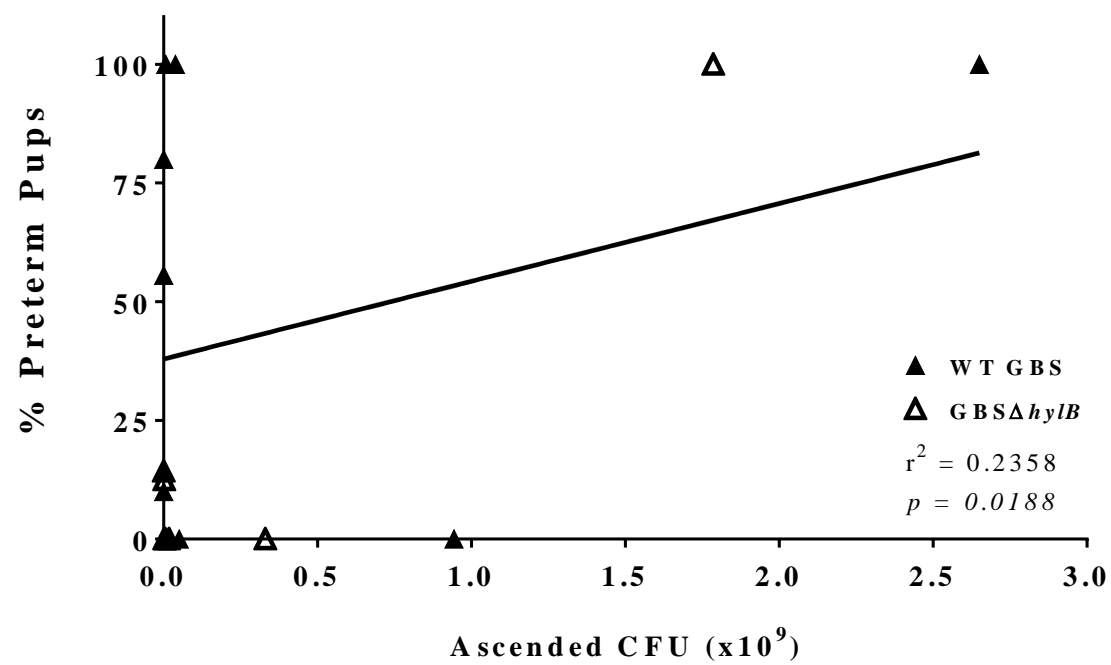

Supplement: Figure S1 — Correlation between ascended GBS and preterm birth. Shown are Spearman’s correlation between the number of ascended GBS (average CFU in uterine space, placentas, and pups) and the percentage of preterm pups (either in the cage or IUFD). Download [file mbo003162877sf1.pdf]

Figure S2

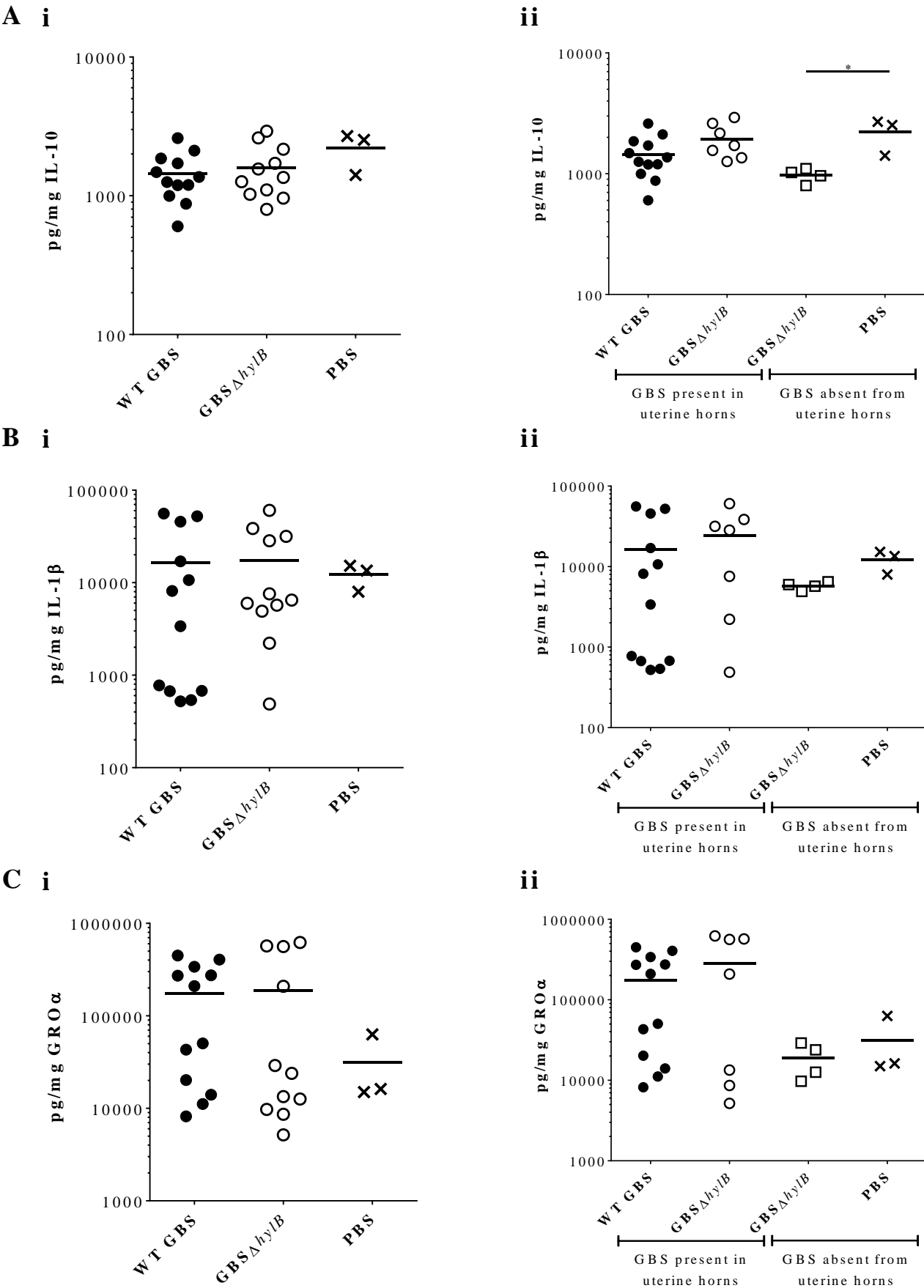

Supplement: Figure S2 — Inflammatory markers not affected by HylB in the uterine space. Luminex assays were used to assess the levels of the inflammatory markers IL-10 (A), IL-1β (B), and GROα (C) in the uterine tissues of pregnant female C57BL/6J mice inoculated with approximately 108 CFU of COH1 (n = 12), COH1ΔhylB (n = 11), or PBS (n = 3). Data are shown from all 3 groups of uterine samples (Ai to Di), uterine samples with GBS present, or uterine samples without GBS (either inoculated with GBS or PBS [Aii to Dii]). Unpaired Student’s t test was used to assess statistical significance between groups (A to D [*, P > 0.05]). Download [file mbo003162877sf2.pdf]

Figure S3

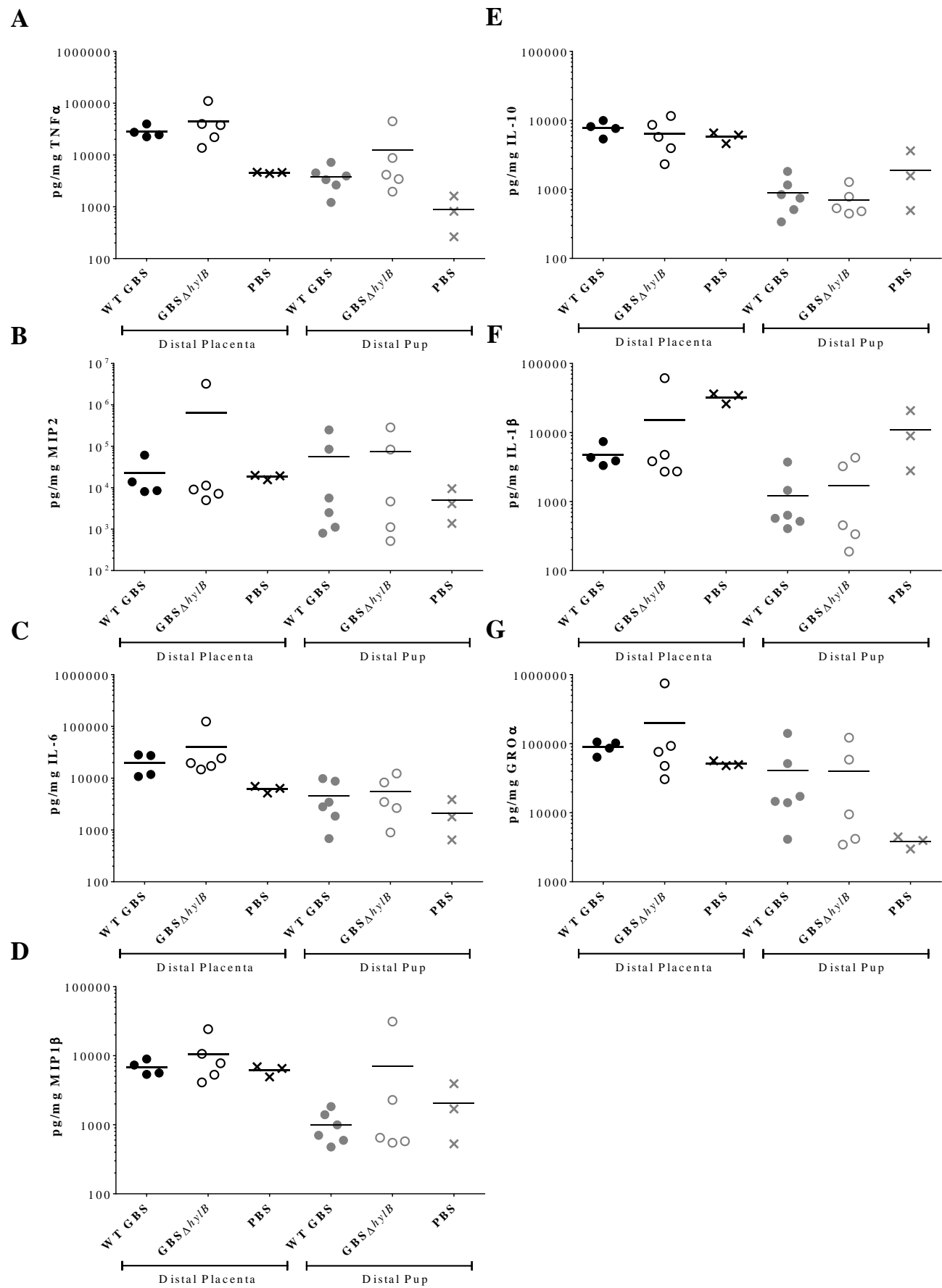

Supplement: Figure S3 — Inflammation not affected by HylB in distal placental or distal pup tissues. Luminex assays were used to assess the levels of the inflammatory markers TNF-α (A), MIP2 (B), IL-6 (C), MIP1β (D), IL-10 (E), IL-1β (F), and GROα (G) in the distal placental tissues (see Fig. 2B for schematic) of pregnant female C57BL/6J mice inoculated with approximately 108 CFU of COH1 (n = 5), COH1ΔhylB (n = 5), or PBS (n = 3). Download [file mbo003162877sf3.pdf]

Figure S4

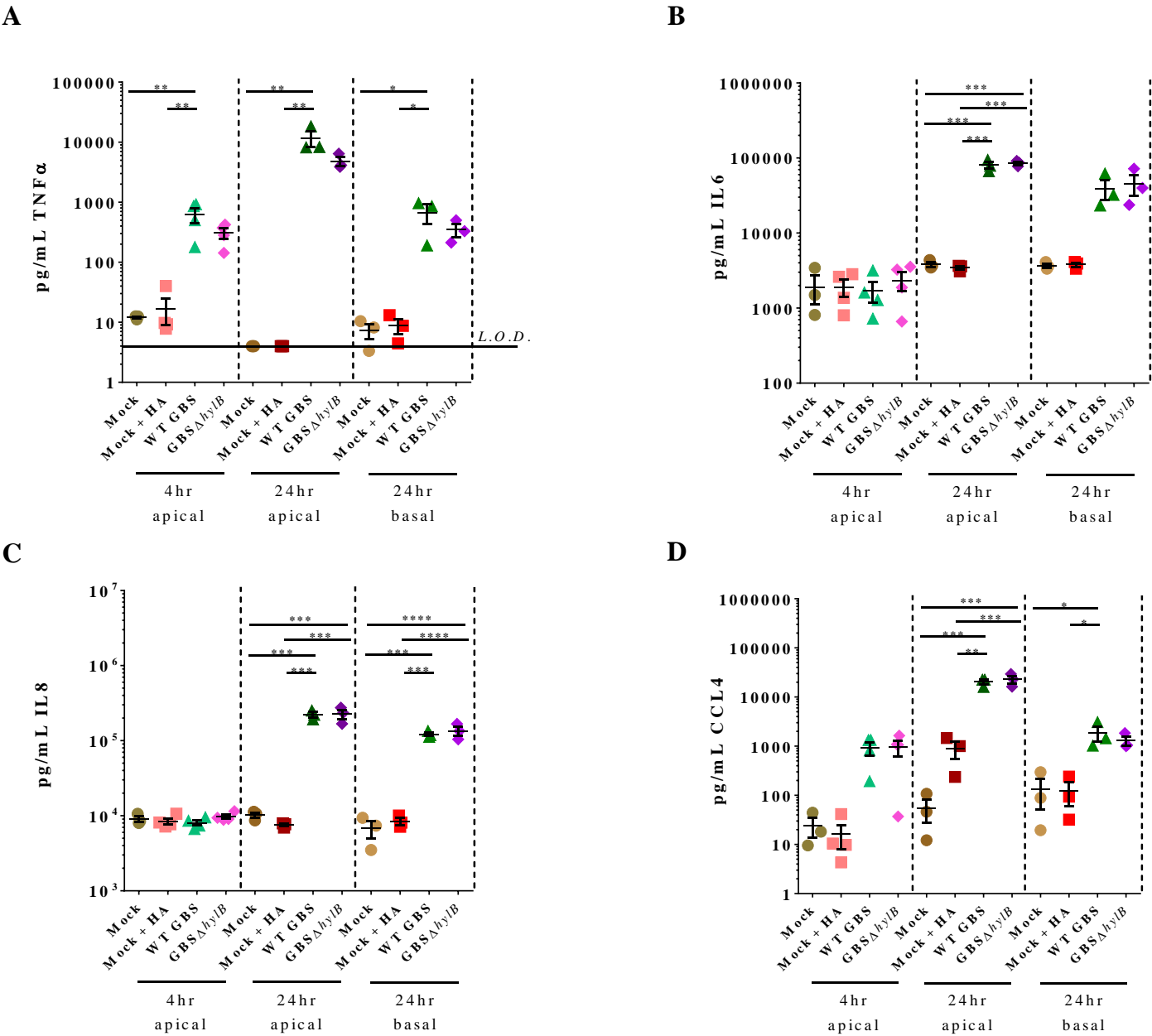

Supplement: Figure S4 — Inflammation not affected by HylB in ex vivo gestational membranes. ELISAs were used to assess the levels of the inflammatory markers TNF-α (A), IL-6 (B), IL-8 (C), CCL4 (D) in the gestational tissues inoculated with approximately 107 CFU of COH1 or COH1ΔhylB. Culture medium was supplemented with 1.25 mg/ml hyaluronic acid (HA). Experiments were performed in duplicate on at least 3 gestational tissues. One-way ANOVA with Tukey’s multiple correction test was used to assess statistical significance between groups (A to D [*, P > 0.05; **, P > 0.005; ***, P > 0.0005; ****, P > 0.00005]). LOD, limit of detection. Download [file mbo003162877sf4.pdf]
